# A novel liquid-liquid phase separation-related gene signature for predicting prognosis in colon cancer

**DOI:** 10.3389/fimmu.2024.1514613

**Published:** 2024-12-19

**Authors:** Shuo Wang, Sen Hou, Shan Jiang, Chao Wang, Peipei Zhang, Yingjiang Ye, Zhidong Gao

**Affiliations:** ^1^ Department of Gastroenterological Surgery, Peking University People’s Hospital, Beijing, China; ^2^ Laboratory of Surgical Oncology, Peking University People’s Hospital, Beijing, China; ^3^ Department of Gastroenterology, Shandong Provincial Hospital Affiliated to Shandong First Medical University, Jinan, Shandong, China; ^4^ Key Laboratory for Neuroscience, Ministry of Education/National Health and Family Planning Commission, Department of Biochemistry, School of Basic Medical Sciences, Peking University Health Science Center, Beijing, China

**Keywords:** liquid-liquid phase separation, colon cancer, prognosis, immunotherapy, nomogram

## Abstract

**Background:**

An increasing body of evidence indicates that dysregulation of liquid-liquid phase separation (LLPS) in cellular processes is implicated in the development of diverse tumors. Nevertheless, the association between LLPS and the prognosis, as well as the tumor immune microenvironment, in individuals with colon cancer remains poorly understood.

**Methods:**

We conducted a comprehensive evaluation of the LLPS cluster in 1010 colon cancer samples from the TCGA and GEO databases, utilizing the expression profiles of LLPS-related prognostic differentially expressed genes (DEGs). Subsequently, a LLPS-related gene signature was constructed to calculate the LLPS-related risk score (LRRS) for each individual patient.

**Results:**

Two LLPS subtypes were identified. Substantial variations were observed between the two LLPS subtypes in terms of prognosis, pathway activity, clinicopathological characteristics, and immune characteristics. Patients with high LRRS exhibited worse prognosis and poorer response to immunotherapy. LRRS was found to be correlated with the clinicopathological characteristics, genomic alterations, and the potential response to immune checkpoint inhibitors therapy of colon cancer patients. Additionally, the biological function of a key gene POU4F1 was verified *in vitro*.

**Conclusions:**

This study highlights the crucial role of LLPS in colon cancer, LRRS can be used to predict the prognosis of colon cancer patients and aid in the identification of more effective immunotherapy strategies.

## 1 Introduction

Colorectal cancer ranks third for cancer incidence and second for cancer mortality globally (1). While advancements in surgery, chemotherapy, and immunotherapy have extended patient survival, the challenge of recurrence and metastasis persists (2). Consequently, there is an urgent necessity to continuously develop novel prognostic models for precise risk stratification and enhance treatment efficacy.

In addition to membrane-bound organelles, such as mitochondria, nucleus, and endoplasmic reticulum, cells also contain a substantial quantity of liquid-like membraneless organelles that function to compartmentalize proteins and nucleic acids, enabling the performance of specialized biological processes (3, 4). The formation of these membraneless organelles relies on liquid-liquid phase separation (LLPS), which allows for swift exchange of components with the adjacent cellular matrix or other organelles due to the absence of membranes, thereby contributing to the maintenance of a relatively stable intracellular environment (5, 6).

The intricate process of membraneless organelle formation involves the coordination of scaffolds, regulators, and clients (7, 8). Initially, scaffolds establish the structural framework, followed by the involvement of clients, while regulators play a crucial role in maintaining the proper functioning of membraneless organelles. The detrimental effects of aberrant LLPS exhibited by proteins such as TDP-43, FUS, and Tau in neurodegenerative diseases have been extensively validated (9–12). Recent studies have also highlighted the significant role of LLPS in the onset and progression of diverse cancers (13–15). For instance, the transcription co-activators YAP and TAZ regulate the transcriptional process during tumor advancement through LLPS. Pathological fusion of genes leads to aberrant occurrences or pathological loss of LLPS, thereby promoting tumor progression (16, 17).

In light of these findings, this study aims to collect gene expression data and clinical information from the TCGA-COAD and GEO cohorts, with the objective of constructing an innovative prognostic model based on LLPS gene expression patterns of these colon cancer patients. Ultimately, the colon cancer patients were divided into two LLPS subtypes exhibiting distinct prognosis, clinicopathological characteristics and tumor immune microenvironments. This study, for the first time, established a LLPS-related gene signature in colon cancer to quantify the LLPS levels in individual patients. This novel model holds promise for facilitating personalized prognosis prediction and better treatment choices for colon cancer patients.

## 2 Materials and methods

### 2.1 Overall flowchart

**Figure 1:**
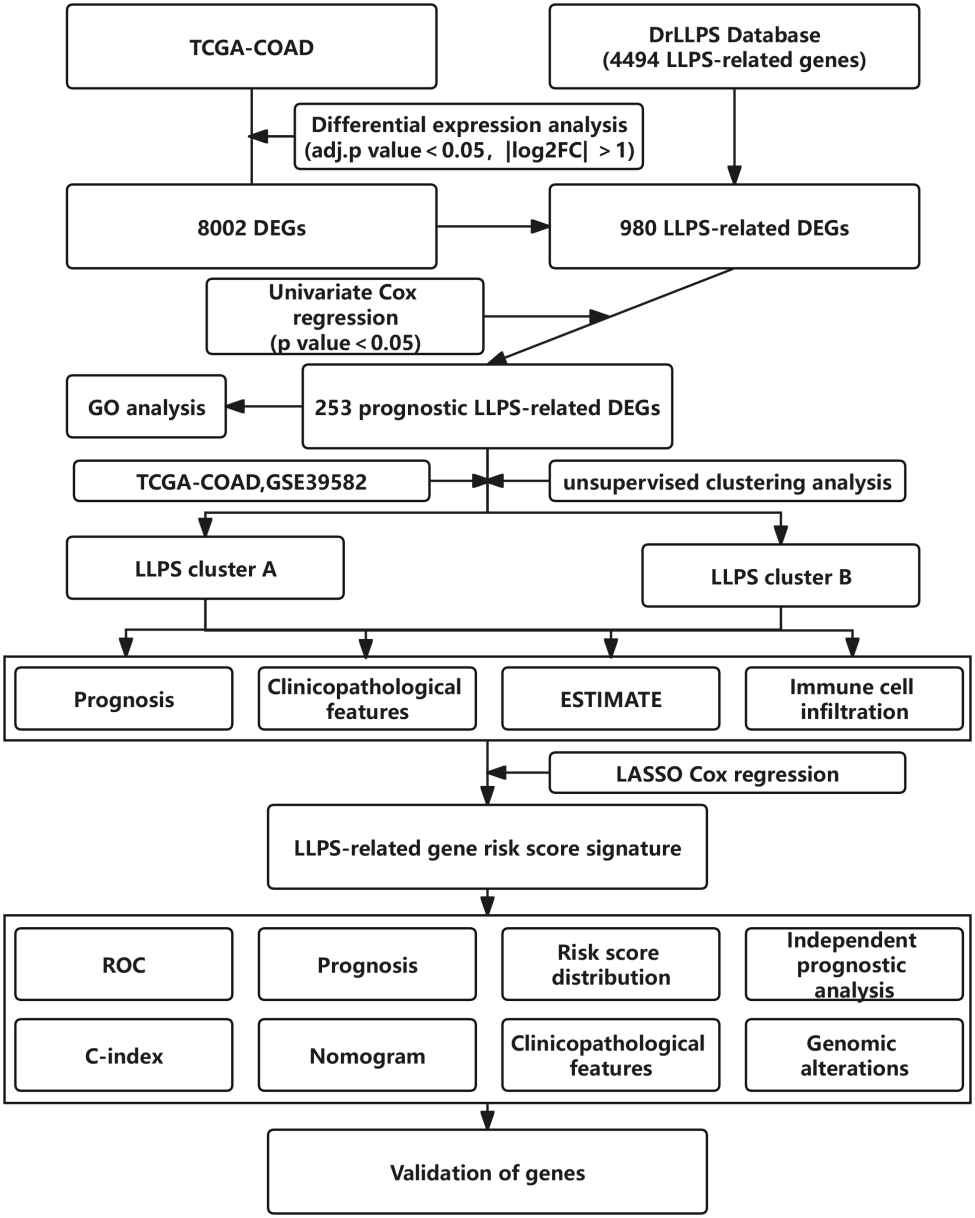
The overall flow diagram of this study.

The overall flowchart of this study was shown in **Figure 1**. Firstly, we screened out the differentially expressed genes (DEGs) between tumor and normal tissues in the TCGA-COAD cohort, followed by intersecting with LLPS-related genes obtained from DrLLPS database. Then, prognostic LLPS-related DEGs were identified by univariate Cox regression. Based on the expression profiles of these genes, unsupervised clustering analysis was performed to identify different LLPS patterns in colon cancer patients. Subsequently, we investigated the heterogeneity of two LLPS subtypes. In addition, the Least Absolute Shrinkage Selection Operator (LASSO) Cox regression is used to construct LLPS-related gene signature to calculate the LRRS. The robustness of signature is evaluated by multiple dimensions.

### 2.2 Datasets and preprocessing

RNA sequencing gene expression profile (including count and TPM value), somatic mutation and clinical information of TCGA-COAD cohort were downloaded from the Cancer Genome Atlas (TCGA) database (https://portal.gdc.cancer.gov/). The count value was used to identify the DEGs between tumor and normal tissues via “limma” R package (18). The normalized series matrix file of GSE39582 was directly downloaded from GEO database (https://www.ncbi.nlm.nih.gov/geo/) (19). The “ComBat” function of the “sva” R package was used to correct the batch effects of non-biological technical biases of TCGA-COAD TPM values and GSE39582 expression data. Patients without survival data were excluded from further analyses (20). Following the exclusion of normal tissue samples and data lacking overall survival (OS) information, the subsequent analysis was conducted on 448 samples from the TCGA-COAD cohort and 562 samples from the GSE39582 cohort. Data were analyzed with R (version 4.1.0).

### 2.3 Source of LLPS-related gene data

A total of 4494 LLPS-related genes, of which 90 were scaffolds (2.00%), 487 were regulators (10.84%), and 3917 were clients (87.16%) (**Supplementary Table S1**) in Homo sapiens involving 36 condensates were obtained from the DrLLPS database (http://llps.biocuckoo.cn/), which is a comprehensive database containing 437887 LLPS related proteins from 164 eukaryotes (21).

### 2.4 Unsupervised clustering identification of LLPS subtypes of colon cancer patients

Firstly, the empirical Bayesian method of “limma” R package was used to identify DEGs between tumor and normal tissues in the TCGA-COAD cohort based on the screening criteria of P<0.05 and |log2FC|>1 (22). Then, intersect with 4494 LLPS-related genes, followed by univariate Cox regression analysis. A total of 253 prognostic LLPS-related DEGs were identified. Based on the expression of 253 prognostic LLPS-related DEGs mentioned above, unsupervised clustering analysis was performed using the “ConsensuClusterPlus” package to identify different subtypes of LLPS in colon cancer patients (23).

### 2.5 Gene set variation analysis

GSVA is a non-parametric, unsupervised method for estimating variation of gene set enrichment through the samples of an expression data set (24). To investigate the differences in Kyoto Encyclopedia of Genes and Genomes (KEGG) signaling pathways among different LLPS subtypes, we conducted GSVA enrichment analysis using the “GSVA” R package (24). The gene set “c2. cp. kegg. v2023.1. Hs. symbols” was download from the MSigDB database (https://www.gsea-msigdb.org/gsea/msigdb). Adjusted P-value less than 0.05 is considered statistically significant.

### 2.6 Functional enrichment

Gene Ontology (GO) annotates genes to three categories including biological processes, molecular functions, and cellular components (25). GO enrichment analysis was performed using the “enrichGO” function of the “clusterProfiler” R package (26).

### 2.7 Evaluation of immune cell infiltration and immune function

Single Sample Gene Set Enrichment Analysis (ssGSEA), which is an extension of Gene Set Enrichment Analysis (GSEA), is used to assess the relative abundance of various immune cell infiltrations and immune functions in patients with colon cancer. Two gene sets, containing 23 and 29 types of immune cell markers respectively (**Supplementary Tables S2**, **S3**), are employed to evaluate the tumor microenvironment and immune functions of different LLPS subtypes in colon cancer (27, 28). The immune scores, stromal scores, ESTIMATE scores, and tumor purity of colon cancer patients was calculated by the “estimate” R package (29). The xCELL, TIMER, quanTIseq, EPIC, ConsensusTME, and ABIS methods from the “immunedeconv” R package are utilized to evaluate the correlation between the LRRS and immune cell infiltration (30). Expression levels of 45 immune checkpoint-related genes were analyzed between LLPS clusters (31).

### 2.8 Prediction of immune checkpoint inhibitor therapy response

The Tumor Immune Dysfunction and Exclusion (TIDE) algorithm (http://tide.dfci.harvard.edu/) was used to evaluate the tumor immune scape potential of colon cancer patients from their expression profiles (32, 33). Patients with lower TIDE scores was more likely to show stronger responses to immune therapy. The immunophenotypic score (IPS) data was download from The Cancer Immunome Atlas (TCIA) database (https://tcia.at/home). Then compared the IPS between different LLPS clusters to evaluate the responsiveness to anti-PD-1 or anti-CTLA-4 therapy (34).

### 2.9 Construction and validation of a LLPS-related gene signature

To construct a LLPS-related gene signature, 253 prognostic LLPS-related DEGs were used to build a LASSO Cox regression model. LRRS was calculated as follows:


$$\text{LRRS}=\sum_{\text{i}=1}^{n}\left(\text{Coe}{\text{f}}_{\text{i}}*\;\text{Ex}{\text{p}}_{\text{i}}\right)$$

where Coef_i_ and Exp_i_ represent the LASSO coefficient and the corresponding gene expression level, respectively. Patients with colon cancer were stratified into low and high LRRS groups according to the median value of LRRS. Univariate and multivariate Cox regression analyses were conducted on LRRS in conjunction with clinical characteristics to identify independent prognostic factors. A nomogram was constructed utilizing these independent prognostic factors. Receiver operating characteristic (ROC) curve analysis were applied to assess the accuracy of nomogram, LRRS, and clinical characteristics in predicting OS of colon cancer patients. The concordance index (C-index) was employed to assess the prognostic capability of nomogram, LRRS, and clinical characteristics. The calibration curves were used to evaluate the precision of the nomogram in terms of the agreement between the observed and predicted OS outcomes at the 1st, 3rd, and 5th years.

### 2.10 Analyses of genomic alterations

The mutation profiles and frequencies were visualized using the “maftools” R package. The tumor mutation burden (TMB) was defined as the number of mutations per megabase (mut/Mb) (35). The copy number variation (CNV) data was acquired from UCSC Xena (https://xena.ucsc.edu/) database. Then identify the copy number amplifications or deletions of model gene in the cohort.

### 2.11 Cell culture

HCT-8 cells were purchased from Wuhan Pricella Biotechnology Co., Ltd. The cells were cultured in RPMI-1640 medium (Gibco, USA) supplement with 10% Fetal Bovine Serum (FBS, Invitrogen Corporation, USA) and 1% penicillin/streptomycin (Gibco, USA) and incubated in humidified incubator containing 5% CO2 at 37 °C.

### 2.12 Lentivirus transfection

Cells (5 × 10^5^ cells/well) were seeded in the six-well plate. After cell attachment, lentivirus carrying overexpression plasmid was added to the culture medium, supplemented with polybrene to reach a final concentration of 1 µg/mL. The lentivirus was removed and replaced with normal growth medium at 12 h transfection. 48 h after transfection, 1 μg/mL puromycin was added to screen for stable cells for one week.

### 2.13 Quantitative real-time polymerase chain reaction

Total RNA was extracted using RNA Extraction Reagent TRIzol (Invitrogen, USA). Then RNA concentration was determined with a spectrophotometer. The RNA was further reverse-transcribed to cDNA using cDNA synthesis kit (Bio-Rad, USA). cDNA was amplified with SYBR Green Supermix (Bio-Rad, USA). The 2^−ΔΔCt^ method (ΔΔCT = ΔCt control−ΔCt sample) was used to calculate the amplification fold. Primer sequences are shown in **Table 1**.

**Table 1:** Primer sequences.

Gene	Forward	Reverse
POU4F1	GAGCACCACCATTATTACCACCTC	AACACGCAGACAGAACAACTAGC
GAPDH	GTCTCCTCTGACTTCAACAGCG	ACCACCCTGTTGCTGTAGCCAA

### 2.14 CCK8 assay

The cells were seeded at 1×10^5^ cells/100 μl in 96 well plates (100 μL/well) and incubated for 24 hours. Add 10 μL of CCK-8 solution (Dojindo, China) to each well and incubate at 37°C for 1-4 hours. The absorbance was determined at 450 nm using the absorbance microplate reader (Bio-Rad, USA).

### 2.15 Clone formation assay

Cell suspension was diluted to the final concentration of 10^3^ cells/mL. Then the cells were seeded into six-well plates (1 mL/well) and incubate for 10 days. After the clones were formed, the cells were fixed with 4% paraformaldehyde for 30 min and stained with crystal violet staining solution for 20 min. Finally, the cells were washed with PBS and photographed. The experiment was repeated at least three times.

### 2.16 Wound healing assay

5x10^5^ cells were seeded in each well of a six-well plate and when the cells were grown to 90% confluency, a straight line was scratched across the cell monolayer with a 200 μL pipette tip. Then, add medium containing 1% serum to each well after washed with PBS. Cell migration was observed and photographed at 0 h and 24 h under the microscope. The experiment was repeated at least three times.

### 2.17 Transwell assay

2 × 10^4^ cells were added to the upper chamber with serum-free medium (Gibco, USA). 600 μL of complete medium was added to the lower chamber. After 24 hours of incubation, the cells in the upper chamber were removed with cotton swabs, and the cells on the lower surface of the chamber were fixed with 4% paraformaldehyde for 30 minutes, and then stained with crystal violet for 30 minutes. Finally, five visual fields were randomly selected to be photographed with the microscope (Olympus, Japan).

### 2.18 Statistical analysis

The Kruskal-Wilcoxon test was used to compare statistical differences between two groups. While statistical differences among more than two groups was compared using the Kruskal-Wallis test. The correlation between expression of model genes was assessed through the Spearman correlation analysis. The survival curves for the prognostic analysis were generated via the Kaplan-Meier method and the significance of differences were identified by log-rank tests. All statistical p value were two-side, with p < 0.05 as statistically significance. All data processing was done in R 4.1.0 software.

## 3 Results

### 3.1 Identification of LLPS subtypes in colon cancer patients based on prognostic LLPS-related differentially expressed genes

**Figure 2:**
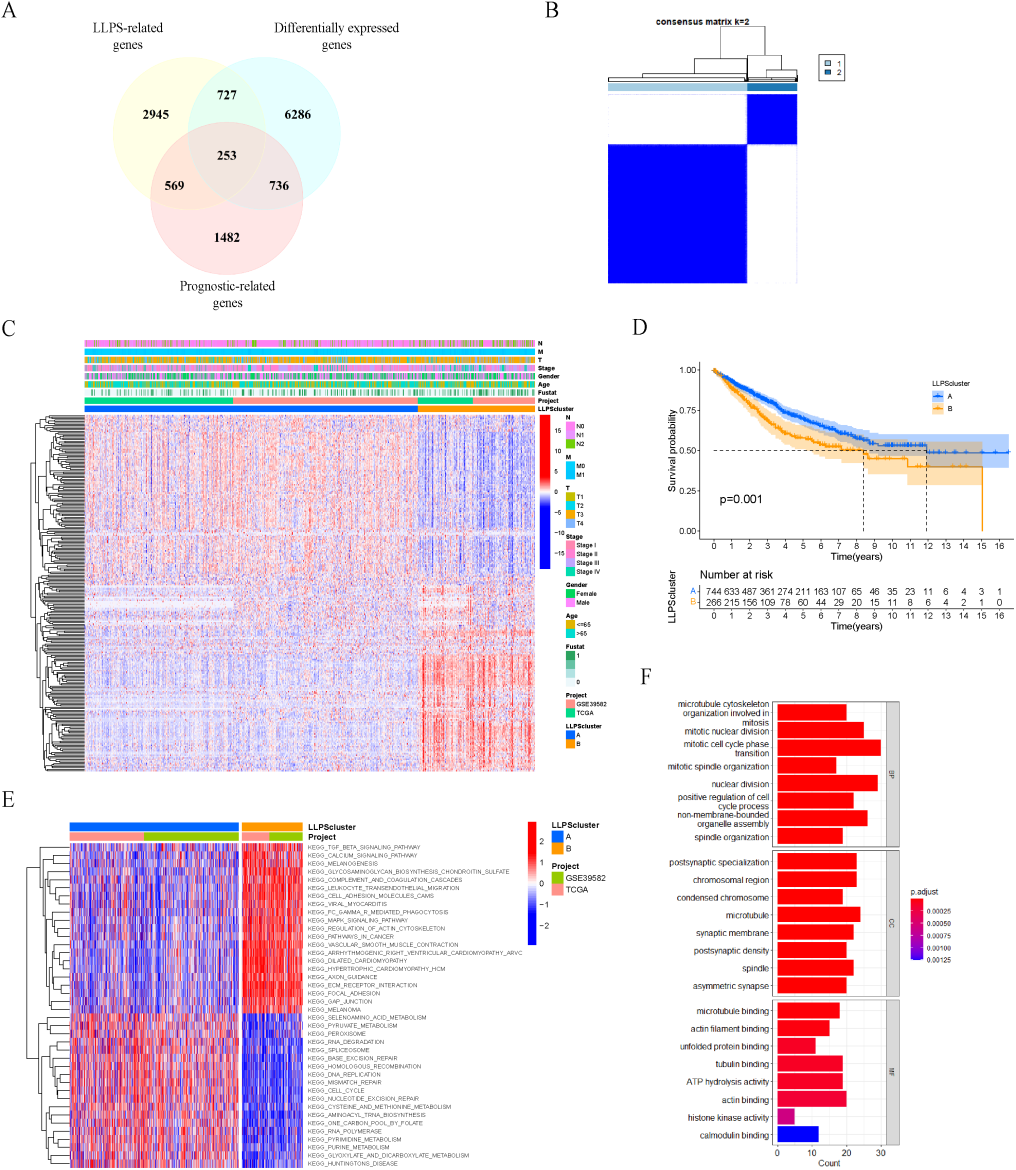
Identification of LLPS subtypes in colon cancer patients based on prognostic LLPS-related differentially expressed genes. **(A)** Venn diagram identified 253 prognostic LLPS-related DEGs in colon cancer. **(B)** Consensus matrix of unsupervised clustering of the TCGA-COAD and GSE39582 cohorts for k = 2. **(C)** Heatmap showed the expression levels of 253 prognostic LLPS-related DEGs among LLPS subtypes. **(D)** Survival curves of OS in two LLPS clusters based on 1010 colon cancer patients from the TCGA-COAD and GSE39582 cohorts. **(E)** Heatmap of KEGG gene sets by GSVA. **(F)** GO enrichment analyses for 253 prognosis LLPS-related DEGs.

To identify the LLPS-related DEGs, we conducted the following screening. Firstly, according to the screening criteria of adj. p<0.05 and | log2FC |>1, 8002 DEGs between tumor samples and normal samples were obtained from the TCGA-COAD cohort. Among them, 3584 were upregulated and 4418 were downregulated in tumor samples compared with normal samples (**Supplementary Table S4**). Subsequently, after intersecting with 4494 LLPS-related genes, 980 LLPS-related DEGs were identified, of which 444 were upregulated and 536 were downregulated in tumor samples (**Supplementary Table S5**). Then, these LLPS-related DEGs were subjected to univariate Cox regression analysis to identify 253 prognostic LLPS-related DEGs (**Figure 2A**), of which 3 were scaffolds, 26 were regulators, and 224 were clients (**Supplementary Table S6**).

Based on the expression levels of the 253 prognostic LLPS-related DEGs mentioned above, we performed unsupervised clustering analysis on the merged TCGA-COAD and GSE39582 cohorts, and ultimately divided 1010 colon cancer patients into two subtypes: LLPS cluster A (n=744) and cluster B (n=266) (**Figure 2B**). The significant differences in the expression of 253 prognosis LLPS-related DEGs between the two subtypes were observed in the heatmap (**Figure 2C**). Prognostic analysis indicated a significant survival difference between the two subtypes of LLPS. Cluster A had a better overall survival outcome than cluster B (**Figure 2D**).

To explore the potential molecular mechanisms underlying the LLPS subtype of colon cancer, we performed GSVA to evaluate the differential KEGG gene sets between the two subtypes. The results showed that cluster A exhibited associations with cell cycle, DNA replication and mismatch repair, whereas cluster B demonstrated associations with the MAPK signaling pathway and JAK-STAT signaling pathway (**Figure 2E**). Additionally, we performed GO enrichment analyses on 253 prognosis LLPS-related DEGs (**Figure 2F**).

### 3.2 Clinicopathological and immune characteristics between different LLPS subtypes

**Figure 3:**
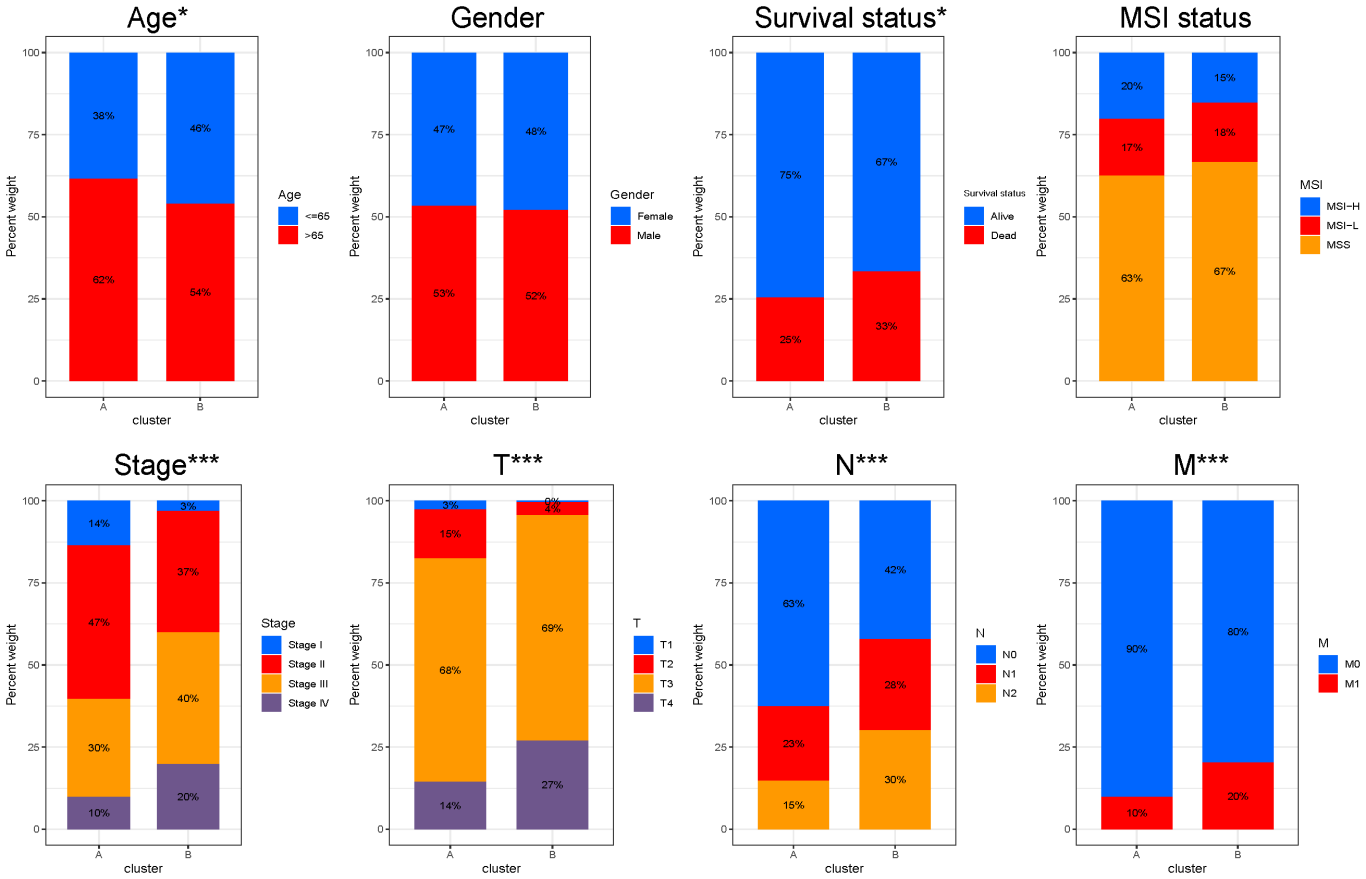
Clinicopathological characteristics between different LLPS clusters. Comparisons of age, gender, survival status, MSI status, stage, T, N and M stage among LLPS subtypes. *P < 0.05, ***P < 0.001.

We attempted to compare the clinicopathological characteristics of colon cancer patients in two different LLPS clusters. Compared to cluster B, cluster A had more patients with age greater than 65 years and higher proportion of stage I and stage II, which may explain why the overall survival outcome of cluster A is better. No statistically significant variances were observed between the two clusters in terms of gender and MSI status distribution (**Figure 3**).

**Figure 4:**
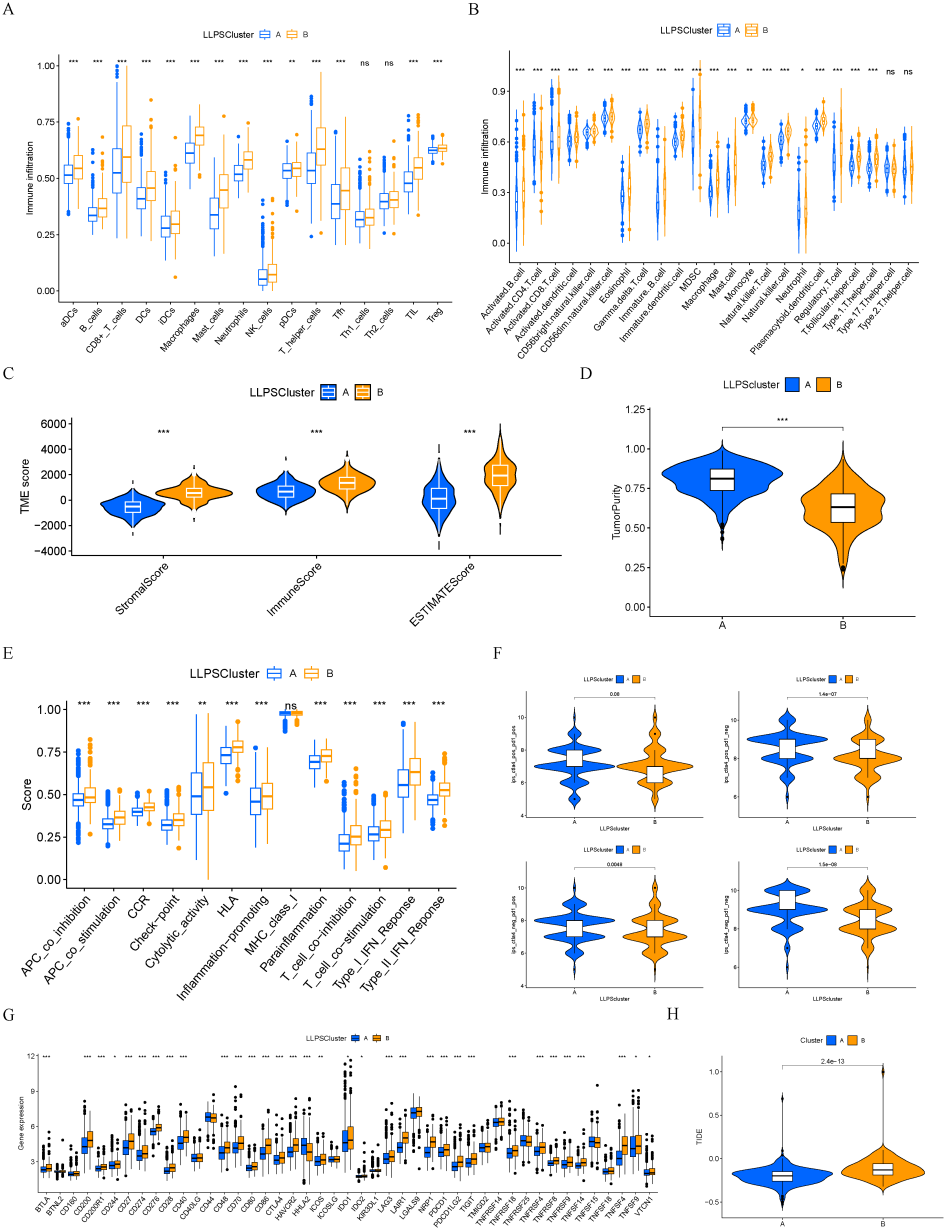
Immune characteristics between different LLPS clusters. **(A, B)** The levels of immune cell infiltrations in two LLPS clusters. **(C)** Comparisons of immune, stromal, and ESTIMATE scores among two LLPS clusters. **(D)** Comparison of tumor purity among two LLPS clusters. **(E)** The scores of immune functions between two LLPS clusters. **(F)** Comparisons of IPS between two LLPS clusters. **(G)** Expression levels of immune checkpoint genes between two LLPS clusters. **(H)** TIDE score between two LLPS clusters. *P < 0.05, **P < 0.01, ***P < 0.001. ns, not significant.

Subsequently, we conducted a comparative analysis of immune cell infiltration among the two subtypes of LLPS. Results derived from two distinct gene sets consistently indicated that cluster B exhibited a higher degree of immune cell infiltration (**Figures 4A, B**). Employing the ESTIMATE algorithm, we predicted the abundance of stromal and immune cells across different subtypes. The analysis revealed that cluster A exhibited lower ESTIMATE, immune, and stromal scores compared to cluster B, indirectly reflecting a higher tumor purity in cluster A (**Figures 4C, D**). Then, we compared the immune functions between the two LLPS subtypes. Notably, cluster B demonstrated significantly elevated functions, particularly in aspects such as immune check-point (**Figure 4E**). A detailed differential analysis highlighted that the expression levels of key immune checkpoint molecules, including CD274 (PD-L1), CTLA4, LAG3, and TIGIT, were markedly higher in cluster B compared to cluster A (**Figure 4F**). Finally, we compared the IPS between the two LLPS clusters. Cluster A demonstrated a significantly elevated IPS, suggesting a more promising immunotherapeutic response potential (**Figure 4G**). TIDE analysis indicated that cluster A had a lower TIDE score, further corroborating its enhanced likelihood of responding favorably to immunotherapeutic interventions (**Figure 4H**).

### 3.3 Construction and evaluation of the LLPS-related gene signature

**Figure 5:**
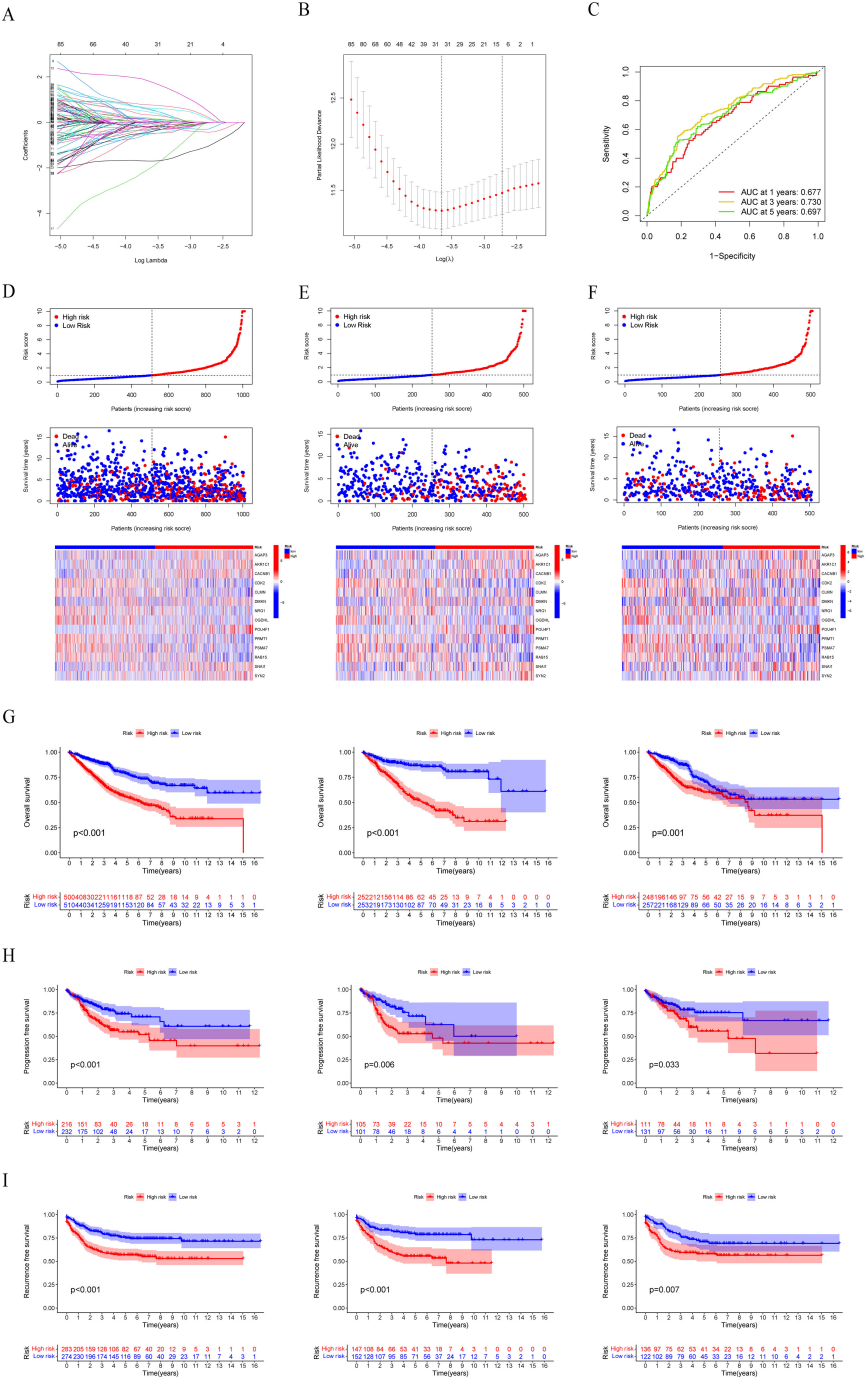
Construction and evaluation of the LLPS-related gene signature. **(A, B)** LASSO Cox regression analysis to construct a LLPS-related gene signature. **(C)** Time-dependent ROC curve analysis in the total group. **(D–F)** The distribution of LRRS, survival time, status, and heatmaps of risk model genes among high- and low-risk patients in the total, train, and test groups. **(G)** Survival curves of OS in the total, train, and test groups. **(H)** Survival curves of PFS in the total, train, and test groups in the TCGA-COAD cohort. **(I)** Survival curves of RFS in the total, train, and test groups in the GSE39582 cohort.

Next, 253 prognostic LLPS-related DEGs were subjected to LASSO Cox regression to construct a LLPS-related gene signature for predicting the prognosis of colon cancer. According to the ratio of 1:1, 1010 colon cancer patients were randomly divided into a train group and a test group. The detailed clinical information of the train group, test group and total group is shown in **Table 2**. Ultimately, 14 genes were identified (**Figures 5A, B**), comprising 7 protective genes and 7 risk genes, with their respective risk coefficients detailed in **Table 3**. The LRRS of each colon cancer patient can be calculated by summing the product of the expression levels of each gene and the corresponding risk coefficients. The area under the curve (AUC) values were evaluated by ROC curve. The LRRS had the highest AUC value in the 3rd year, reaching 0.730. Additionally, AUC values of 0.677 and 0.697 were observed in the first and fifth years, respectively (**Figure 5C**). According to the median value of LRRS, colon cancer patients in the train and test group were divided into high and low risk groups, respectively. The distribution of LRRS, survival time, status, and expression heatmaps of risk model genes among high- and low-risk patients in the total, train, and test groups were displayed (**Figures 5D–F**). The prognostic analysis of the overall survival time of CRC patients revealed that those with high LLPS scores exhibited poorer prognoses in all the three groups (**Figure 5G**). Furthermore, a comparative analysis of progression free survival in the TCGA-COAD cohort (**Figure 5H**) and recurrence-free survival in the GSE39582 cohort (**Figure 5I**) demonstrated that individuals with higher LRRS experienced inferior prognoses in the total, train and test groups.

**Table 2:** Clinical information of total, train and test groups.

Covariates	Total	Train	Test	P Value
Age				0.6346
<=65	406(40.2%)	199(39.41%)	207(40.99%)	
>65	603(59.7%)	306(60.59%)	297(58.81%)	
unknown	1(0.1%)	0(0%)	1(0.2%)	
Gender				0.6136
Female	467(46.24%)	229(45.35%)	238(47.13%)	
Male	543(53.76%)	276(54.65%)	267(52.87%)	
Stage				0.31
Stage 0	4(0.4%)	1(0.2%)	3(0.59%)	
Stage I	107(10.59%)	55(10.89%)	52(10.3%)	
Stage II	438(43.37%)	226(44.75%)	212(41.98%)	
Stage III	328(32.48%)	164(32.48%)	164(32.48%)	
Stage IV	122(12.08%)	51(10.1%)	71(14.06%)	
unknown	11(1.09%)	8(1.58%)	3(0.59%)	
T				0.2114
Tis	4(0.4%)	1(0.2%)	3(0.59%)	
T1	21(2.08%)	14(2.77%)	7(1.39%)	
T2	120(11.88%)	62(12.28%)	58(11.49%)	
T3	669(66.24%)	326(64.55%)	343(67.92%)	
T4	175(17.33%)	97(19.21%)	78(15.45%)	
unknown	21(2.08%)	5(0.99%)	16(3.17%)	
N				0.5609
N0	565(55.94%)	291(57.62%)	274(54.26%)	
N1	235(23.27%)	122(24.16%)	113(22.38%)	
N2	178(17.62%)	84(16.63%)	94(18.61%)	
unknown	32(3.17%)	8(1.58%)	24(4.75%)	
M				0.0919
M0	879(87.03%)	447(88.51%)	432(85.54%)	
M1	123(12.18%)	52(10.3%)	71(14.06%)	
unknown	8(0.79%)	6(1.19%)	2(0.4%)	

**Table 3:** LASSO coefficients of 14 LLPS-related risk genes.

Gene	coefficients
AGAP3	1.636
AKR1C1	1.107
CACNB1	1.514
CDK2	-2.137
CLMN	1.620
DMKN	0.446
NRG1	-1.501
OGDHL	-0.787
POU4F1	2.759
PRMT1	-4.322
PSMA7	-2.110
RAB15	1.962
SNAI1	1.189
SYN2	1.101

### 3.4 Development and evaluation of nomogram

**Figure 6:**
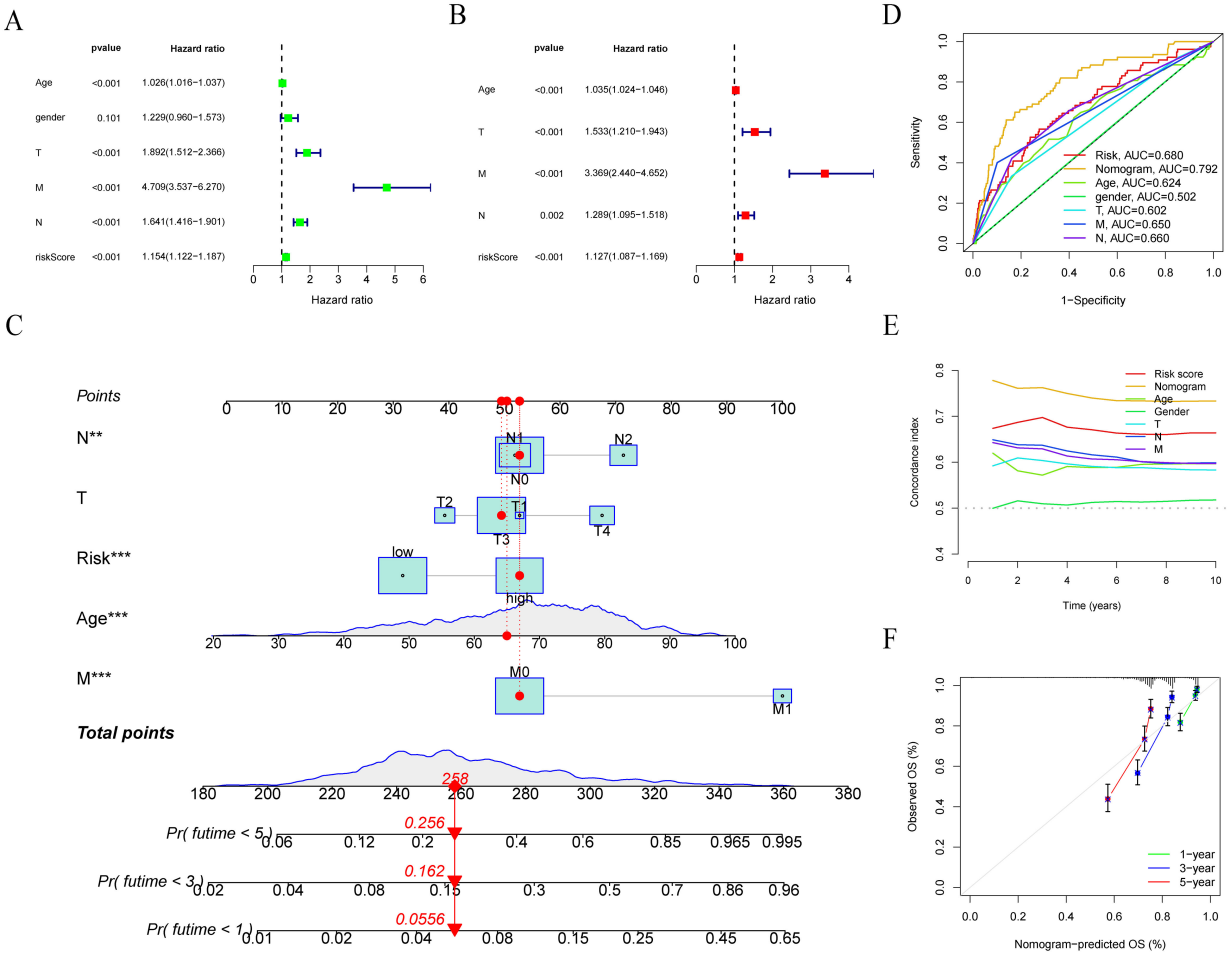
Development and evaluation of nomograms. **(A, B)** Univariate and multivariate Cox regression analyses of LRRS and clinical information with overall survival. **(C)** Nomogram was developed by integrating age, TNM stage, and LLPS risk. **(D)** ROC curve analysis of nomogram, LRRS and clinical information. **(E)** Concordance index of LRRS and clinical information. **(F)** Calibration plots to assess the accuracy of nomogram. **P < 0.01, ***P < 0.001.

We want to build a survival prediction model for colon cancer patients that can be applied in clinical practice. To this end, we first conducted univariate and multivariate Cox regression analyses of LRRS and clinical information with overall survival. The results indicated that the LRRS is an independent prognostic factor for OS. In univariate Cox regression analyses, the hazard ratio (HR) of LRRS was 1.154 with a 95% confidence interval (CI) of 1.122-1.187 (p<0.001, **Figure 6A**). In multivariate Cox regression analyses, the HR of LRRS was 1.127 with a 95% CI of 1.087-1.169 (p<0.001, **Figure 6B**). In addition, age and TNM stage were also independent prognostic factors. Subsequently, a nomogram was developed by integrating age, TNM stage, and LLPS risk (**Figure 6C**). By calculating the score of each variable, the 1-, 3-, and 5-years survival of colon cancer patients can easily estimate by drawing a vertical line. The ROC curve demonstrated that the LRRS had excellent accuracy in predicting OS, with an AUC of 0.680, surpassing the predictive ability of any other clinical feature. Furthermore, the nomogram showed an even higher AUC of 0.792 (**Figure 6D**). Meanwhile, the concordance index indicated that the nomogram had the highest predictive accuracy, followed by LRRS (**Figure 6E**). The calibration curves also illustrate that there is a good agreement between the observed and predicted OS outcomes at the 1st, 3rd, and 5th years for the nomogram (**Figure 6F**).

### 3.5 Clinicopathological and immune characteristics between the two LRRS groups

**Figure 7:**
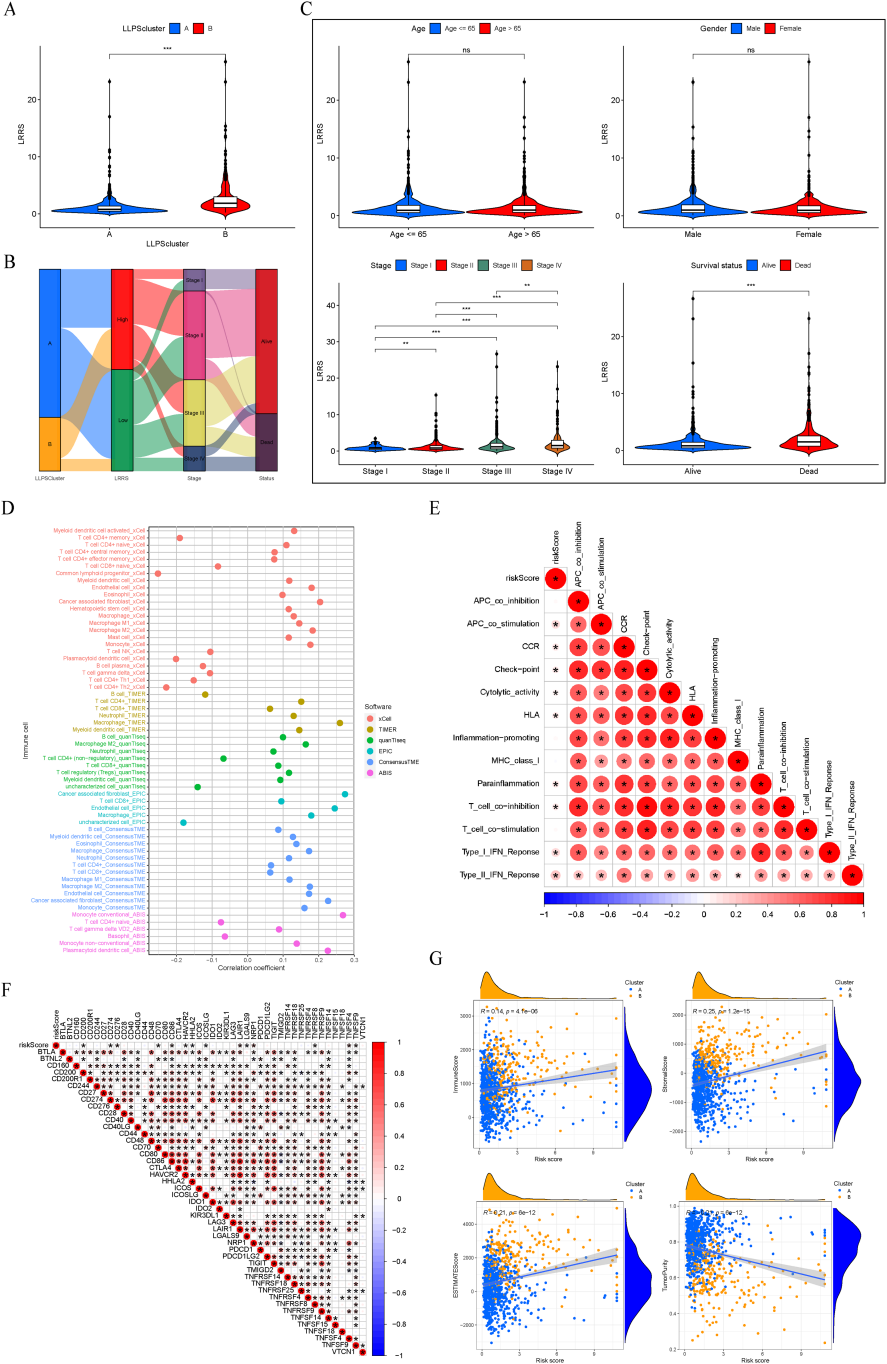
Clinicopathological and immune characteristics between the two LRRS groups. **(A)** Cluster B exhibited a significantly higher LRRS compared to cluster **(A, B)** Sankey diagram of LLPS clusters, LRRS, clinical stage, and survival status. **(C)** Comparisons of age, gender, clinical stage, and survival status between the high and low LRRS groups. **(D)** Correlation between LRRS and immune cell infiltration. **(E)** Correlation between LRRS and immune function. **(F)** Correlation between LRRS and expression levels of immune checkpoint genes. **(G)** Correlation between LRRS and immune, stromal, ESTIMATE scores, and tumor purity. *P < 0.05, **P < 0.01, ***P < 0.001. ns, not significant.

We conducted a comparison of the LRRS among various LLPS clusters, revealing that cluster B exhibited a significantly higher LRRS compared to cluster A (**Figure 7A**). The relationship between LLPS clusters, LRRS, clinical stage, and survival status is shown in **Figure 7B**. Notably, a majority of patients within Cluster B were categorized into the high LRRS group, consistent with the results in **Figure 7A**. Then, the Wilcoxon test was used to calculate the correlation between LRRS and clinicopathological features including age, gender, clinical stage, and survival status. The results showed that high LRRS was associated with high clinical stage and poor prognosis, but not with age and gender. The LRRS gradually increased from stage I to stage IV (**Figure 7C**).

Subsequently, employing a panel of methodologies including xCELL, TIMER, quanTIseq, EPIC, ConsensusTME, and ABIS, we investigated the correlation between LRRS and immune cell infiltration. The findings indicated that T cell NK, macrophage, dendritic cell, cancer associated fibroblast, and monocyte were positively correlated with LRRS, while basophil and T cell CD4+ memory were negatively correlated with LRRS (**Figure 7D**). An immune functional correlation analysis revealed that LRRS was positively correlated with immune functions such as immune checkpoint and T cell co-inhibition (**Figure 7E**). Consistently, LRRS was positively correlated with the expression of immune inhibitory molecules such as CD274, LAIR1, and NRP1 (**Figure 7F**). In our final analysis, we assessed the relationship between LRRS and comprehensive immunological scores, including immune, stromal, ESTIMATE scores, and tumor purity. The results demonstrated that LRRS was positively correlated with immune, stromal, and ESTIMATE scores, but negatively correlated with tumor purity (**Figure 7G**). In summary, these analyses underscore that while the high LRRS group shows enhanced immune cell infiltration, it paradoxically exhibits a higher state of immune suppression.

### 3.6 Genomic alterations of two LRRS groups

**Figure 8:**
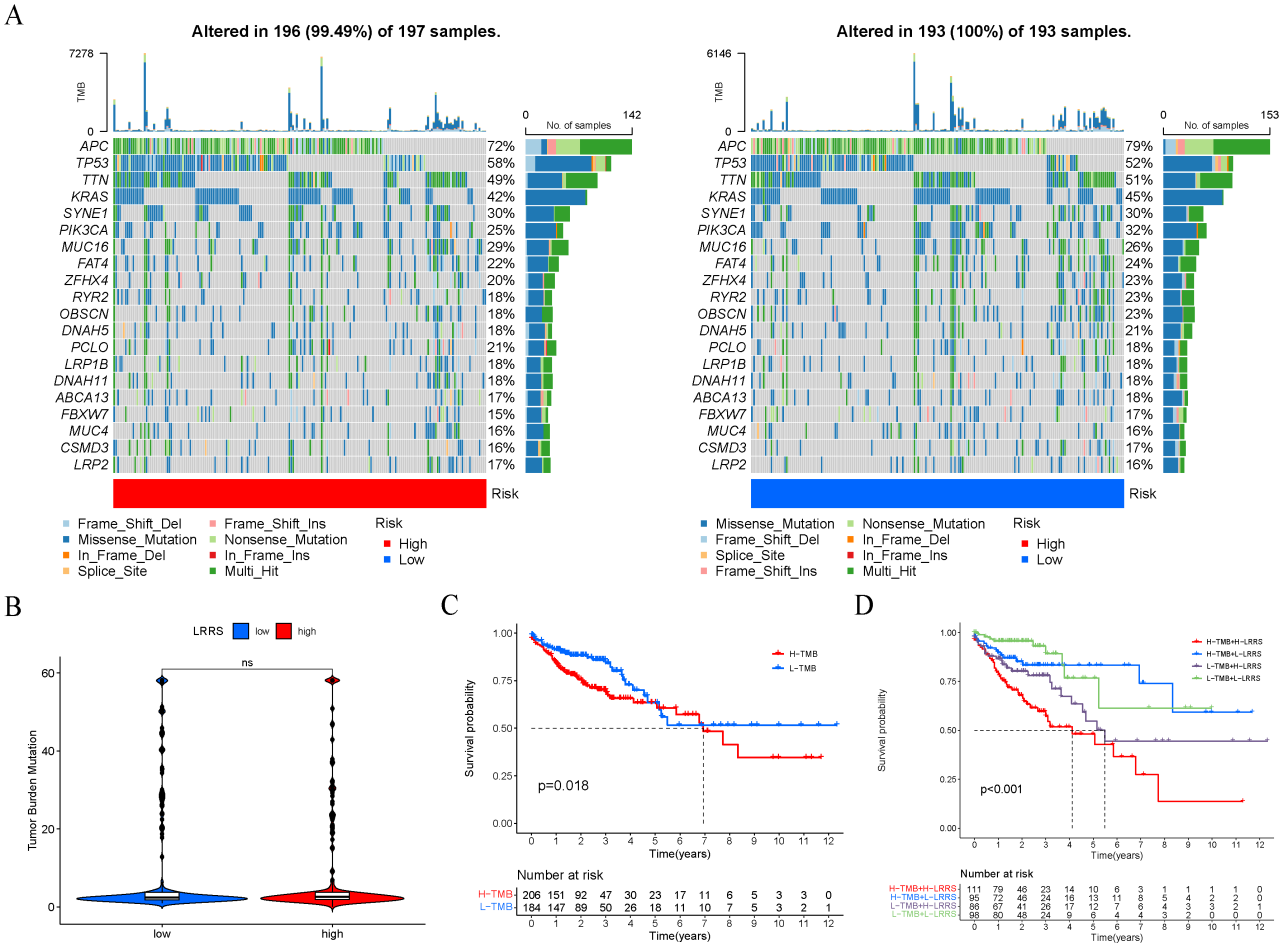
Genomic alterations of two LRRS groups. **(A)** The mutation frequency of the top 20 genes in the high and low LRRS group. **(B)** The TMB value showed no significant difference between the high and low LRRS group. **(C)** Survival curves of OS in high and low TMB groups. **(D)** Survival curves of OS in four groups (high-TMB + high-LRRS, high-TMB + low-LRRS, low-TMB + high-LRRS, low-TMB + low-LRRS). ns, not significant.

Next, we analyzed the top 20 mutated genes in the two different groups (**Figure 8A**). The gene mutation frequencies were found to be similar between the two groups. APC, TP53, and TTN are the three genes with the highest mutation frequency. Additionally, the TMB was evaluated between the high and low LRRS groups, revealing no significant difference in TMB levels (**Figure 8B**). However, survival analysis showed that the prognosis of the high TMB group was significantly worse than that of the low TMB group (**Figure 8C**). Therefore, we wonder whether it is possible to combine TMB and LRRS to stratify patients, and the results showed that this combination could better predict patient prognosis. The group with high TMB and high LRRS had the worst prognosis, followed by the group with low TMB and high LRRS (**Figure 8D**).

### 3.7 Landscape of LLPS-related risk genes in colon cancer

**Figure 9:**
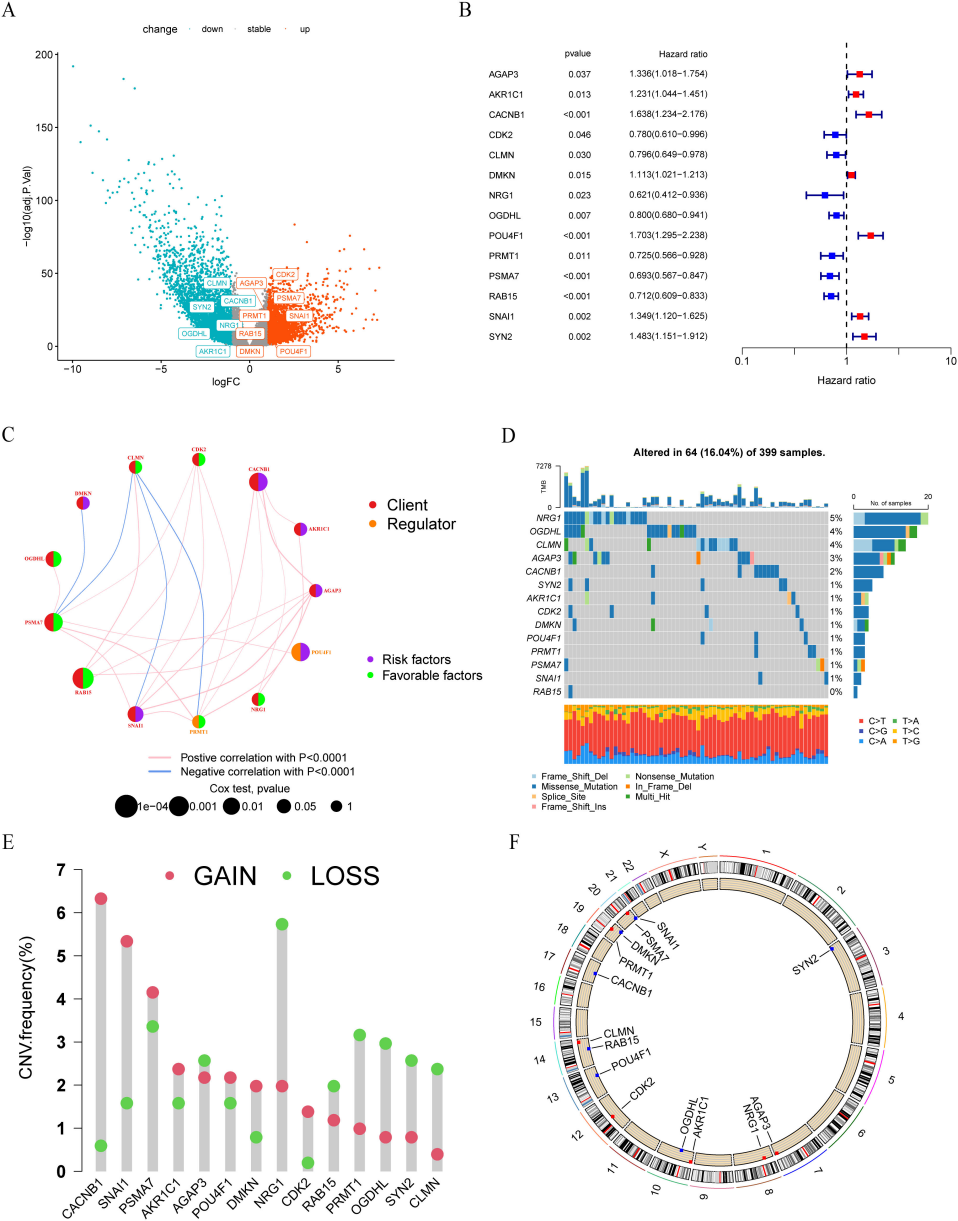
Landscape of LLPS-related risk genes in colon cancer. **(A)** Volcano plot of DEGs between tumor and normal tissues in TCGA-COAD cohort. **(B)** Univariate COX regression analysis of the hazard ratio between14 model genes and overall survival in TCGA-COAD cohort. **(C)** The interaction between 14 model genes in colon cancer. **(D)** The mutation frequency of 14 model genes in 399 colon cancer patients from TCGA-COAD cohort. **(E)** The CNV variation frequency of 14 model genes in TCGA-COAD cohort. **(F)** The location of CNV alteration of 14 model genes on 23 chromosomes.

A total of 14 genes were utilized in the construction of the LLPS-related gene signature. Among these genes, AGAP3, CDK2, DMKN, PRMT1, PSMA7, POU4F1, RAB15 and SNAI1 were up-regulated in tumor tissue compared with normal tissue in TCGA-COAD cohort, while the remaining genes were down-regulated in tumor tissue (**Figure 9A**). Hazard ratios (95% CI) of these genes were calculated by univariate Cox hazard analysis (**Figure 9B**). In terms of gene types, these 14 genes consisted of 11 clients, 2 regulators, and 1 scaffold. Except for the scaffold SYN2, there existed an expression correlation among other genes (**Figure 9C**). Next, we summarized the incidence of somatic mutations of 14 LLPS-related genes in TCGA-COAD cohort. Among 399 tumor samples, only 64 (16.04%) exhibited mutations in the model genes. The gene with the highest mutation frequency was NRG1, but only 5%, indicating that these LLPS-related model genes are conserved in the progression of colon cancer (**Figure 9D**). The statistical results of CNV alteration frequency showed that the CNV alteration was prevalent in 14 model genes. Specifically, copy number amplification was more pronounced in CACNB1, CDK2, DMKN, and SNAI1, while CLMN, NRG1, OGDHL, PRMT1, and SYN2 mainly exhibit copy number deletion, consistent with their mRNA expression (**Figure 9E**). The location of CNV changes of these genes on chromosomes was shown in **Figure 9F**.

### 3.8 POU4F1 promoted proliferation and migration of HCT-8 cells

**Figure 10:**
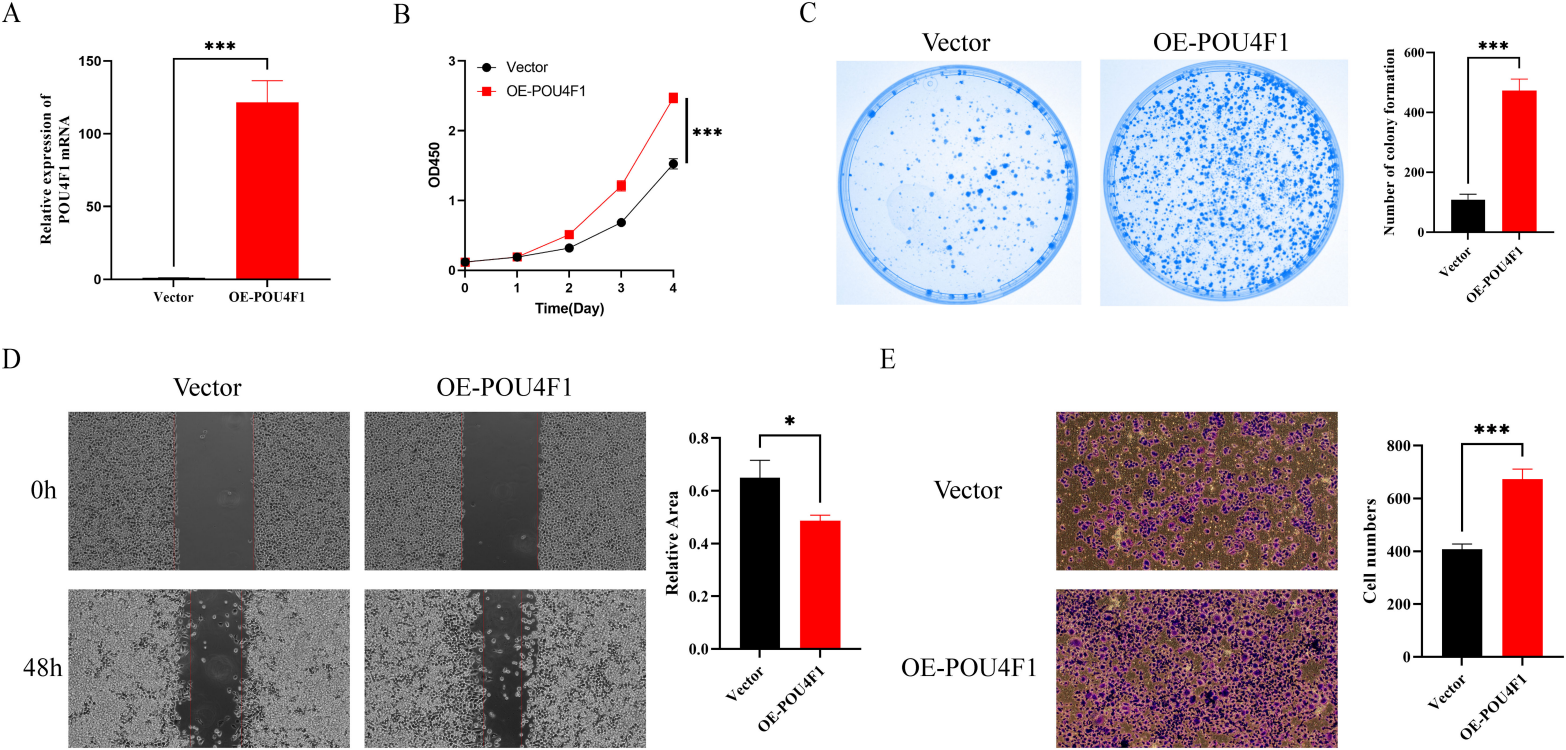
POU4F1 promoted proliferation and migration of HCT-8 cells. **(A)** qRT–PCR analysis of POU4F1 in OE-POU4F1 and control groups. **(B)** Assessment of the proliferation of HCT-8 cells transfected with OE-POU4F1 and vector by CCK8 assay. **(C)** Representative images and quantitative analysis of clone formation in OE-POU4F1 and vector cells. **(D)** Representative images and quantitative analysis of wound healing assay of HCT-8 cells transfected with OE-POU4F1 and vector. **(E)** Assessment of the migration of HCT-8 cells transfected with OE-POU4F1 and vector by Transwell assay. Error bars denote means ± standard deviation (SD). n = 3 biological repeats for each group. OE-POU4F1: POU4F1 overexpression group; vector: control group; *P < 0.05, ***P < 0.001.

Given that POU4F1 is a regulator which may play a key role in the LLPS process, we conducted an overexpression study of POU4F1 in HCT-8 cells to elucidate its effects on cell proliferation and migration. POU4F1 overexpression were achieved by lentivirus transfection (**Figure 10A**). Further investigations were conducted to determine the proliferation and migration of HCT-8 cells. The CCK8 assay showed cell proliferation increased after POU4F1 overexpression (**Figure 10B**). Meanwhile, an elevated number of clones were observed followed by POU4F1 upregulation (**Figure 10C**). Subsequently, wound healing assay was performed and the results showed that the overexpression of POU4F1 resulted in a significant enhancement of the cell migration (**Figure 10D**). Transwell migration assay also showed that the number of migrated cells in the POU4F1-overexpressing group was greater than that in the control group (**Figure 10E**). The findings above suggested that POU4F1 facilitated both proliferation and migration of HCT-8 cells.

## 4 Discussion

Numerous studies have shown that the LLPS process of proteins is closely related to the occurrence and development of various diseases, especially neurodegenerative diseases and tumors. Given that the majority of research has concentrated on the LLPS of single protein in disease progression, a comprehensive exploration of LLPS-associated genes holds significant importance in the identification of novel tumor subtypes and the prediction of prognosis.

In this study, we attempted to explore the role of LLPS-related genes in colon cancer, which has a high incidence and mortality rate in the world. Based on the expression levels of 253 prognostic LLPS-related DEGs, an unsupervised clustering was used to classify 1010 colon cancer patients from the TCGA-COAD and GSE39582 cohorts into two different LLPSS subtypes. The two LLPS subtypes have different prognosis, pathway activity, clinicopathological features, and immune infiltration. To our knowledge, this is the first study to characterize the LLPS-related gene signature in colorectal cancer. In order to better conduct personalized comprehensive evaluation, all 1010 patients were divided into a train group and a test group in a 1:1 ratio. A LLPS-related risk score, namely LRRS including 14 genes was constructed using LASSO Cox regression. By calculating the LRRS for each patient in the cohort, patients were divided into high and low risk groups. LRRS was associated with the prognosis, clinicopathological features and genomic changes of colon cancer patients.

Currently, multiple studies have shown that the LLPS of intracellular molecules can affect tumor progression through various biological processes. The SUMOylated RNF168 catalyzed by SENP1 prevents the occurrence of LLPS, allowing RNF168 to be recruited to DNA damage sites for nonhomologous DNA end-joining, thereby maintaining genomic stability and even making tumor cells resistant to chemotherapy (36). The LLPS of YAP and TAZ compartmentalizes key cofactors to regulate tumor development by activating the transcription of target gene (37, 38). The LLPS of YBX1 enhanced by circASH2 promotes the decay of TPM4 transcripts, effectively inhibiting the metastasis of hepatocellular carcinoma by mediating cytoskeleton remodeling (39). The LLPS of an aberrant chimera NUP98-HOXA9, generated by recurrent chromosomal translocation of NUP98, can bind to and enhance the activation of target genes, promoting the development of acute leukemia (40).

The high LRRS group showed higher levels of immune cell infiltration and immune related functional scores, indicating that it has higher immunogenicity. We attempt to explain this phenomenon from the perspective of genomic alterations. We analyzed the top 20 mutated genes in the high and low LRRS groups. TP53, MUC16 and USH2A have higher mutation frequencies in the high LRRS group, while the mutation frequencies of RYR2 and OBSCN are higher in the low LRRS group. Growing evidence suggests that TP53, one of the most famous tumor suppressor genes in various cancers, contributes to the regulation of tumor immune response (41, 42). It has been found that TP53 mutations can significantly activate the innate immune pathway in CRC (41). MUC16, which encodes the well-known cancer antigen 125 (CA-125), has a very high mutation frequency in multiple tumors. A study involving 10195 patients across 30 solid tumors in the TCGA database showed that MUC16 mutations resulted in higher abundance of immune cells in the tumor microenvironment and increased expression of multiple inhibitory checkpoints (43). The above researches support for the higher immunogenicity in the high LRRS group. It is necessary to conduct further research to investigate the specific mechanism by which these gene mutations regulate the tumor immune microenvironment.

LRRS consists of 14 prognostic LLPS-related differentially expressed genes, including 1 scaffold, 2 regulators and 11 clients. Synaptin II, as a scaffold, is encoded by SYN2, which is one of the three genes encoding synaptic proteins. Synaptin II is involved in droplet and postsynaptic density, playing a crucial role in controlling synapse formation and growth, neuron maturation and renewal, as well as the mobilization, docking, fusion, and recycling of synaptic vesicles (44). In addition, there have been reported that the expression level of SYN2 is significantly related to the prognosis of breast cancer, suggesting that it may play a role (45). The regulator POU4F1, a transcription factor of the POU gene family, is mainly expressed in neuronal cells and can activate the transcriptional activity of the antiapoptotic gene bcl-2 to protect neuronal cells from apoptosis (46). Its role has been validated in breast cancer, melanoma, thyroid cancer and glioma (47–50). Several studies suggest that POU4F1 may be a hub gene in certain signature of colorectal cancer, yet its specific role has not been experimentally validated (51–53). We successfully overexpressed the transcription factor POU4F1 in HCT8 cells and observed a significant enhancement in both cell proliferation and migration. These findings underscore the pivotal role of POU4F1 in the oncogenic process of CRC, thereby broadening our understanding of its contribution to tumorigenesis. However, the particular function of POU4F1 in the LLPS process has not been thoroughly investigated, and we believe this will be an entirely new field. The other regulator PRMT1 is one of the major protein arginine methyltransferases in mammals. Due to the substrate of PRMT1 regulate various biological functions, the dysregulation of arginine methylation caused by PRMT1 may lead to the progression of cancer (54). A total of 6 out of 11 clients can participate in postsynaptic density. Among them, AKR1C1 and OGDHL can also participate in nucleolus. The other two members of Nucleolus are NRG1 and PSMA7. DMKN participates in p-body. CDK2 is quite comprehensive and participates in various membraneless organelles including nucleolus, centrosome/spindle pole body and stress granule. SNAI1 is a unique protein that cannot be classified, and further research is needed to determine the localization of the droplets it forms. Although we understand the types of membraneless organelles involved in these model genes, further exploration is still needed on the specific roles played by some model genes in tumor progression.

Anyway, there are several limitations in our study. First, all analyses were performed based on the retrospective data of TCGA and GEO databases, using prospective data would be more convincing. Second, due to the lack of the treatment-related information in GSE39582 cohort, all of the relevant analyses only used data from TCGA database. Finally, our study did not elucidate the specific molecular mechanisms of model genes in the progression of colon cancer, and further experiments evidence are needed in the future.

## 5 Conclusion

In summary, we divided colon cancer patients into two subtypes of LLPS, which have different prognosis, pathway activity, clinicopathological features and immune cell infiltration. In addition, we constructed an LLPS-related gene signature to predict the prognosis of colon cancer patients. Patients with high LRRS have worse prognosis and poorer response to immunotherapy. Our findings might contribute to personalized prognosis prediction and better treatment options for colon cancer patients, but further studies are needed to confirm this point.

## Data Availability

The datasets presented in this study can be found in online repositories. The names of the repository/repositories and accession number(s) can be found in the article/**Supplementary Material**.
